# Selection for Phytophthora Root Rot Resistance in Chickpea Crosses Affects Yield Potential of Chickpea × *Cicer echinospermum* Backcross Derivatives

**DOI:** 10.3390/plants13111432

**Published:** 2024-05-22

**Authors:** Sean L. Bithell, Muhammd A. Asif, David Backhouse, Andre Drenth, Steve Harden, Kristy Hobson

**Affiliations:** 1 New South Wales Department of Primary Industries, Tamworth, NSW 2340, Australia; 2 Chickpea Breeding Australia, New South Wales Department of Primary Industries, Tamworth Agricultural Institute, Tamworth, NSW 2340, Australia; ahsan.asif@dpi.nsw.gov.au (M.A.A.);; 3 School of Environmental and Rural Sciences, University of New England, Armidale, NSW 2350, Australia; backhoused@gmail.com; 4 Centre for Horticultural Science, University of Queensland, Brisbane, QLD 4072, Australia; a.drenth@uq.edu.au

**Keywords:** partial resistance, root disease, resistance drag, wild relative, 100 seed weight, agronomic traits

## Abstract

Phytophthora root rot (PRR) of chickpea (*Cicer arietinum*) caused by *Phytophthora medicaginis* is an important disease. Partial resistance to PRR is sourced from *Cicer echinospermum*. In this study, we evaluated if lines with low levels of PRR foliage symptoms in two contrasting recombinant inbred line (RIL) populations parented by chickpea cultivars (Yorker and Rupali) and 04067-81-2-1-1 (*C. echinospermum*, interspecific breeding line) had a significant drag on yield parameters. For the Yorker × 04067-81-2-1-1 population with the highest level of PRR resistance, in the absence of PRR, low foliage symptom RIL had significantly later flowering and podding, lower grain yields, and lighter seed and shorter plant phenotypes than high foliage symptom RIL. A quantitative trait locus analysis identified significant QTL for flowering, height, 100-seed weight, and yield, and there was a significantly higher frequency of alleles for the negative agronomic traits (i.e., drag) from the 04067-81-2-1-1 parent in low foliage symptom RIL than in high foliage symptom RIL. For the Rupali × 04067-81-2-1-1 population with lower levels of PRR resistance, in the absence of PRR, low foliage symptom RIL had significantly lighter seed and shorter plants than high foliage symptom RIL. Significant QTL were detected, the majority were for the timing of flowering and podding (n = 18), others were for plant height, yield, and 100-seed weight. For this second population, the frequency of alleles for the negative agronomic traits from the 04067-81-2-1-1 parent did not differ between low and high foliage symptom RIL. The 100 seed weight of RIL under moderate PRR disease pressure showed some promise as a yield component trait to identify phenotypes with both high levels of PRR resistance and grain yield potential for further seed number evaluations. We identified that large population sizes are required to enable selection among chickpea × *C. echinospermum* crosses for high levels of PRR resistance without a significant drag on yield.

## 1. Introduction

Improved disease resistance is required as plant pathogens can decrease productivity and cause significant crop losses that affect food security. Plant pathogens are either directly or indirectly responsible for 20–40% of global annual crop losses [1]. Soon after commercial Australian chickpea (*Cicer arietinum*) production began in the late 1970s, crop losses from Phytophthora root rot (PRR), caused by the Oomycete, *Phytophthora medicaginis*, were recorded [2,3], particularly in north-eastern Australia [4,5]. Metalaxyl seed treatment can provide initial control during crop establishment, but full season PRR protection from cost-effective chemistry has been ineffective, as found for other pulse crops [6]. Ineffective chemical control has led to a focus on resistance breeding to provide a genetic solution to PRR in chickpea [5,7]. Chickpea is susceptible to *P. medicaginis* across all developmental stages [8]. Plant death is earlier in susceptible genotypes than in more resistant genotypes [9,10]. Foliar symptoms, including chlorosis, premature death, or early senescence, are in fact the indirect effects of root damage caused by chickpea [8,9].

Domesticated chickpea varieties do not possess high levels of resistance to *P. medicaginis*. Chickpea plant survival to maturity is a useful parameter of *P. medicaginis* resistance for field-based phenotyping [9,11]. Field and glasshouse studies of chickpea germplasm have identified lines with partial resistance to *P. medicaginis*, but also demonstrated inconsistent responses between the field and glasshouse reactions [10]. Although *C. arietinum*-based chickpea varieties, such as cv. Yorker, have improved PRR variety resistance [12], field-based evaluations have shown that this resistance was not effective under conditions of high disease pressure and in seasons conducive to PRR [13]. Measurement of *P. medicaginis* inoculum development under Yorker in relation to PRR-induced normalized yield losses indicated that the putative basis of improved PRR resistance in Yorker was substantially tolerance based [14]. The absence of effective sources of resistance in chickpea to *P. medicaginis* led to the evaluation of alternative resistance sources, including wild relatives of chickpea [5,15].

Sources of resistance to PRR in chickpea are available but they may also affect other traits. Among five species of wild relatives of chickpea, *Cicer echinospermum* accessions provided the highest level of resistance to *P. medicaginis*, this resistance was also successfully transferred to the progeny of chickpea × *C. echinospermum* crosses [7]. *Cicer echinospermum*-chickpea derivatives have a complex form of quantitative resistance to *P. medicaginis* [11]. A 43.8 Mb region on chromosome 6 was identified as an introgression segment from a *C. echinospermum* backcross (04067-81-2-1-1(B)) used in the Australian chickpea breeding program as a *P. medicaginis* resistance source [16]. However, approximately two thirds of haplotypes with this segment had lower grain yields than those without, this yield effect was considered to be caused by linkage drag [16]. These results indicated that improved selection processes may be required for chickpea × *C. echinospermum* crosses to identify material that has both high yields and PRR resistance. Studies of chickpea × *C. echinospermum* and *C. reticulatum* crosses have demonstrated the transgressive segregation of important agronomic traits, including growth habit, height, days to flowering, 100-seed weight, and grain yield in crosses [17,18,19,20]. Quantitative trait locus (QTL) in chickpea × *C. echinospermum* populations for key yield-related traits have been identified, although no positive alleles for yield traits from the *C. echinospermum* parent were identified [20]. Therefore, it will be important to determine if the alleles of these QTL are effective at identifying high yielding genotypes within the progeny of wild relative crosses used in breeding programs.

Consistent selection or phenotyping across seasons in pathosystems with quantitative resistance can be challenging, as the expression of partial resistance is highly dependent on the prevailing environmental conditions. Genotype by environment interactions involving partial resistance may be due to the differing resistance thresholds among genotypes across a pathogen density gradient resulting in different infection intensities among genotypes [21]. In some chickpea × *C. echinospermum* recombinant inbred lines (RIL), the level of resistance to *P. medicaginis* showed strong environmental interactions [11]. Chickpea × *C. echinospermum* RIL can also contain both tolerance and quantitative resistance to *P. medicaginis*, which may further complicate the expression of disease symptoms and yield loss interpretation among genotypes across environments [14].

The current chickpea breeding objectives in Australia involve finding the most beneficial combination of alleles to achieve high levels of disease resistance with high grain yield and grain quality. Therefore, our overall research objective was to determine if selection for resistance based on foliage symptoms enables the selection of material that maximizes the yield across multiple environments. As high-yielding material can only be selected at maturity, it is also important to determine if the disease assessments and QTLs can be used to improve the selection process for high-yielding resistant lines. We specifically sought to test whether the:

1. phenology and yield traits of chickpea × *C. echinospermum* RILs in the absence of PRR disease differed among genotypes with low and high levels of PRR resistance;
2. foliage symptoms and yields among chickpea × *C. echinospermum* RIL differed across *P. medicaginis* inoculated and natural inoculum experiments;
3. foliage symptom and yield parameters under *P. medicaginis* inoculated and natural inoculum conditions could be used to identify high-resistance RIL with a high yield potential.

An improved understanding of the disease assessment methods, the effects of wild relatives on agronomic traits, and trait composition in the chickpea breeding program is required to maximize sustainable yield and achieve genetic gain for resistance in chickpea against *P. medicaginis*. To test these research questions, we used two F_6_ RIL populations, a cv. Rupali × *C. echinospermum* backcross RIL (hereafter as, RB RIL), and a cv. Yorker × *C. echinospermum* backcross RIL population (hereafter as, YB RIL). The cv. Rupali is very susceptible to *P. medicaginis* while cv. Yorker has tolerance to *P. medicaginis* under low-to-moderate PRR disease pressure [14].

## 2. Results

To identify the potential effects of selection for PRR resistance on yield potential, PRR resistance phenotyping results from 2014 and 2015 *P. medicaginis* inoculated experiments were used to select the groups of RIL with different foliage symptom phenotypes to represent RILs with low and high levels of partial resistance to *P. medicaginis*. The 2014 experiments provided RIL selections (6 high, 6 low) from low disease pressure conditions, whereas the 2015 irrigated experiments provided selections (20 high, 20 low) from high disease pressure conditions.

### 2.1. Non-Diseased Grain Production Experiments

**Figure 1:**
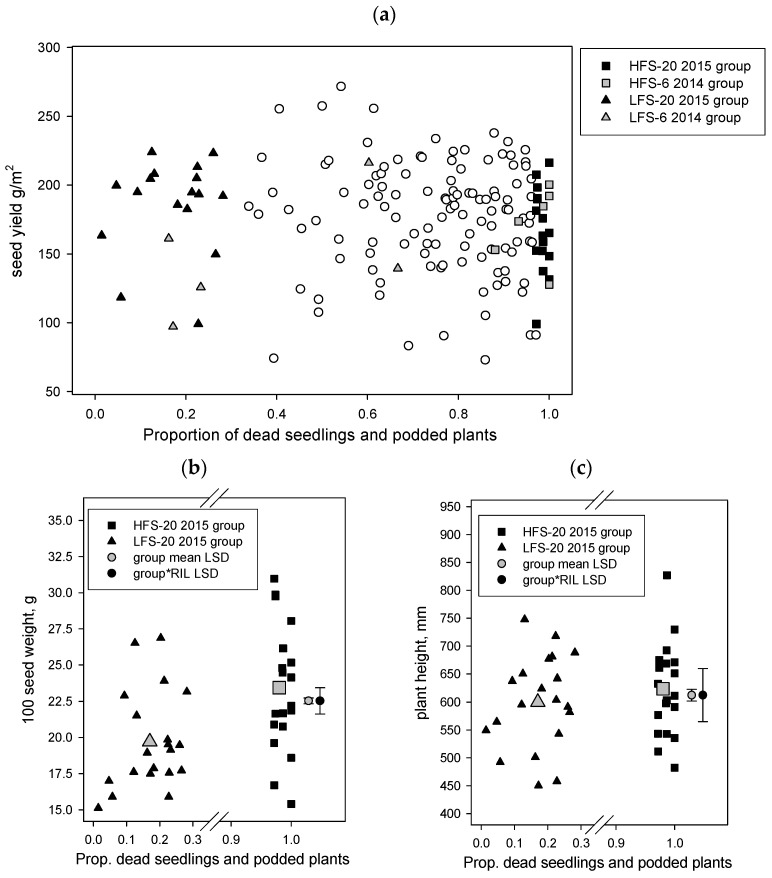
Rupali × *C. echinospermum* backcross RIL population, the proportion of dead seedlings and podded plants from the Hermitage 2015 *Phytophthora medicaginis* inoculated irrigated experiment on x axis versus the results of the Tamworth 2017 non-diseased grain production experiment, y axis results, for the parameters (**a**) whole population seed yield, (**b**) 100-seed weight, and (**c**) plant height. The 2014 6 RIL and 2015 20 RIL foliage symptom phenotype groups (high foliage symptom, HFS; low foliage symptom, LFS) are presented for plot a. For plots b and c only, the 2015 20 RIL foliage symptom phenotype groups are presented and include group means and least significant difference (LSD) values for the main effects and foliage symptom group by RIL interaction.

**Figure 2:**
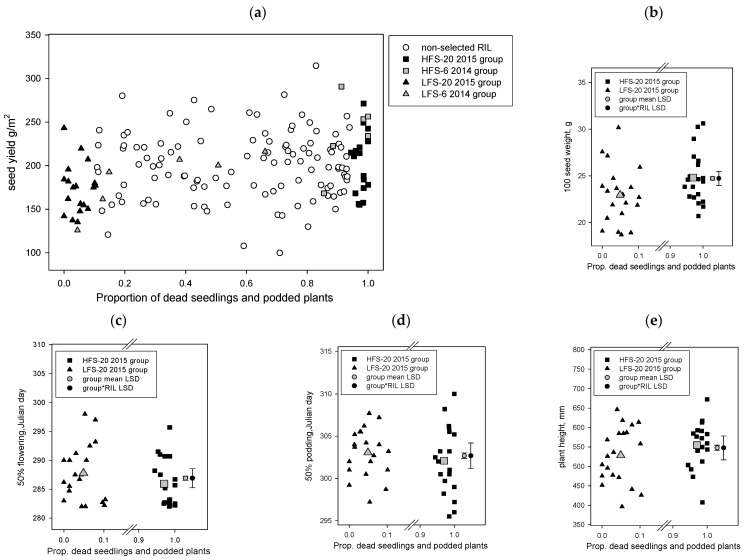
Yorker × *C. echinospermum* backcross RIL population, the proportion of dead seedlings and podded plants from Hermitage 2015 *Phytophthora medicaginis* inoculated irrigated experiment on x axis versus the results of the Tamworth 2017 non-diseased grain production experiment y axis results, for the parameters (**a**) seed yield, (**b**) 100-seed weight, (**c**) 50% flowering day, (**d**) 50% podding day, and (**e**) plant height. The 2014 6 RIL and 2015 20 RIL foliage symptom phenotype groups (high foliage symptom, HFS; low foliage symptom, LFS) are presented for plot a. For plots b to e only, the 2015 20 RIL foliage symptom phenotype groups are presented and include group means and least significant difference (LSD) values for the main effects and foliage symptom group by RIL interaction.

The timing of the phenological stages related to the grain production and grain yields of the two RIL populations in the absence of PRR disease symptoms was evaluated in 2017 to test for the PRR resistance phenotype effects on grain production. Foliage symptom and stem base assessments of PRR susceptible entries during these experiments indicated that there was no PRR disease in either experiment. The trait correlations of all RIL in each population showed that the Julian day of 50% flowering and 50% podding had the highest coefficients (RB 0.75, YB 0.59) in both populations (Supplementary Material, Figure S1). Both populations also showed similar correlations between height and grain yield (0.32–0.33). To compare the non-PRR diseased grain production traits of RIL to their PRR resistance level, data from irrigated *P. medicaginis* inoculated experiments where there was a high PRR disease pressure held in 2015 were used (Figure 1 and Figure 2). The resistance phenotype effects on grain production are presented here for selection set two, as the 2014 low disease pressure conditions led to selections that did not provide a clear segregation among phenotype groups under the 2015 high disease pressure conditions (Figure 1a and Figure 2a). The results for selection set one, six RIL low disease pressure phenotype groups, are provided Supplementary Material S1.

#### 2.1.1. RB RIL Yield Traits between the High and Low Foliage Symptom Phenotypes

Grain yields did not differ significantly (*p* > 0.05) between the low and high disease groups in the RB RIL population (Figure 1a); however, the 100-seed weight did differ significantly (*p* < 0.001, LSD 0.408) where the low foliage symptom (19.7 g) group had a lower weight than the high foliage symptom (23.4 g) group (Figure 1b). There was also a significant interaction (*p* < 0.001, LSD 1.824) between the foliage symptom group and the RIL, whereby a small number of specific RIL had 100 seed weight values significantly smaller or larger than RIL in their foliage symptom group. The 50% flowering and podding dates did not differ significantly (*p* > 0.05) among the low and high disease groups in this population, but again there were also significant interactions (*p* < 0.001) between the foliage symptoms group and RIL for both parameters. Plant height was the only other parameter to differ significantly (*p* < 0.05, LSD 21.23) among groups, with the low foliage symptom (600 mm) group having shorter plants than the high foliage symptom (624 mm) group (Figure 1c). Correlations among the phenology and yield traits across the 40 selected RIL in this selection showed that height was positively and significantly (*p* < 0.001) correlated with both 100-seed weight (0.445) and grain weight (0.408), whereas grain weight was negatively correlated with first flower (−0.371), 50% flowering (−0.417), first pod (−0.303), and 50% podding (−0.354). Hundred-seed weight was not significantly correlated with any phenology parameter. Similar to the whole YB population, this selection set also had a strong correlation (0.74) between the day of 50% flowering and 50% podding.

#### 2.1.2. YB RIL Yield Traits between High and Low Foliage Symptom Phenotypes

The results showed that the yields of selected RIL in the foliage symptom groups were within the range observed for the whole YB RIL population (Figure 2a) and included significant (*p* < 0.001, LSD 12.28) yield differences, with the 20 low foliage symptom group (171.4 g/m^2^) having a lower yield than the 20 high foliage symptom group (208.8 g/m^2^). The results for 100-seed weight also demonstrated similar effects (*p* < 0.001, LSD 0.3404), the low foliage symptom group (22.95 g) had a lower value 100-seed weight than the high foliage symptom group (24.81 g) (Figure 2b). There were also significant interactions between the foliage symptom group and the RIL, where specific RILs within a foliage symptom group had seed yields (*p* < 0.001 LSD 54.93) or 100-seed weight (*p* < 0.001 LSD 1.522) values that were either lower or higher than values for specific RIL in the other foliage symptom group or within the same group (Figure 2a,b). For this population, there were also significant differences (*p* < 0.001, LSD 0.739) in the 50% flowering date among the 20 RIL phenotype groups, the high foliage symptom group had 50% flowering (Julian day 285.9) 1.5 days earlier than the 20 low foliage symptom group (Julian day 287.8) (Figure 2c). Fifty percent pod development was also significantly earlier (*p* = 0.006, LSD 0.675) for the high foliage symptom group (Julian day 302.1) than the low foliage symptom group (Julian day 303.1) (Figure 2d). There were also significant (*p* < 0.001) foliage symptom group by RIL interactions for 50% flowering (LSD 3.304) and 50% percent pod development (LSD 3.018) (Figure 2c,d). Plant height also differed significantly (*p* < 0.001, LSD 13.75) between groups, with the low foliage symptom (529 mm) group having shorter plants than the high foliage symptom (555 mm) group, and included a significant (*p* < 0.001, LSD 61.75) foliage symptom group by RIL interaction (Figure 2e). Correlations among the phenology and yield traits across the 40 selected RIL in this selection showed that height was positively and significantly (*p* < 0.001) correlated with both 100-seed weight (0.315) and grain weight (0.394), height was not significantly correlated with any phenology parameters. Hundred-seed weight was significantly (*p* < 0.001) and negatively correlated (−0.321) with 50% flowering date. Similar to the whole RB population, this selection set also had a strong correlation (0.63) between the day of 50% flowering and 50% podding.

#### 2.1.3. QTL Analysis of Yield and Agronomic Related Traits

**Figure 3:**
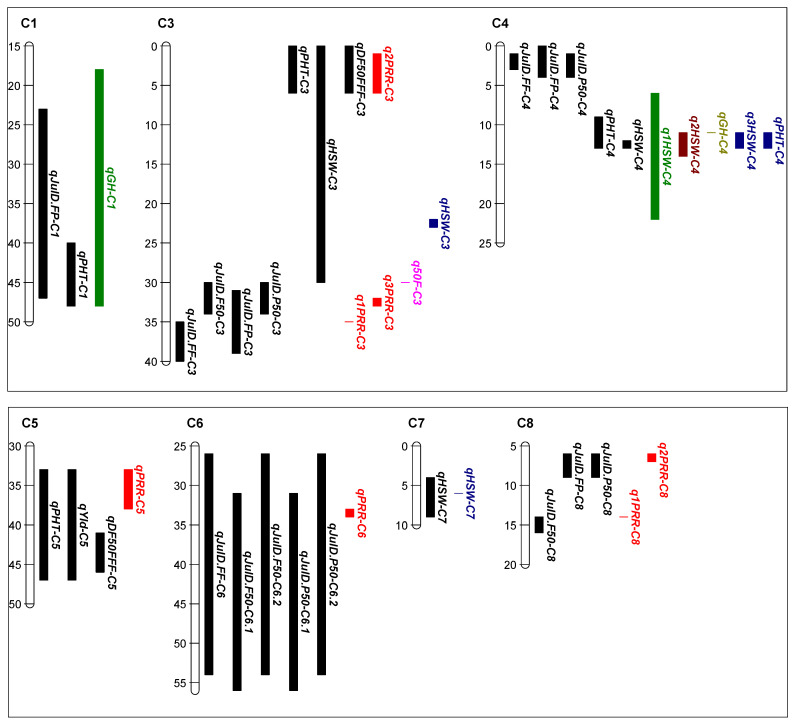
Position of quantitative trait loci (QTL) detected in Rupali × *C. echinospermum* backcross recombinant inbred line (RIL) population (black bars) for the Tamworth 2017 non-diseased grain production experiment. The vertical QTL bars represent the physical position (two LOD drops from the QTL maximum likelihood value). The colored bars represent the QTL identified in previous studies. Growth habit *qGH-C1* [20], *qGH-C4* [22], phytophthora root rot resistance *q1PRR-C3*, *q2PRR-C3*, *q3PRR-C3*, *qPRR-C5*, *qPRR-C6*, *q1PRR-C8*, *q2PRR-C8* [11], 50% flowering *q50F-C3* [23], 100-seed weight *qHSW-C3*, *q3HSW-C4*, *qHSW-C7* [24], *q1HSW-C4* [22], *q2HSW-C4* [25], and plant height *qPHT-C4* [24].

QTL analysis of the RB RIL population: from the whole population QTL analysis, a total of 26 significant QTL were detected which were located on seven chromosomes with LOD scores ranging between 2.8 and 33.2 in RB RIL population (Table 1 a, Supplementary Material Table S1). This included seven QTL for the traits that differed (100 seed weight (n = 4) and plant height (n = 3)) amongst the 20 RIL high disease pressure phenotype groups, four of the seven QTL were associated with negative agronomic effects (three for shorter plants, one for lighter seed) for alleles from the *C. echinospermum* backcross parent (Table 1 a). The majority (n = 18) of the other QTL were related to the timing of flowering and podding, and one QTL for grain yield was identified (Supplementary Material Table S1). The phenotypic variation explained by a single QTL ranged between 5.0% and 52.8%. The physical position of this QTL and the QTLs identified in previous studies in the same genomic regions are shown in Figure 3. A comparison of the frequency of the agronomically positive and negative QTL alleles, as classified in Table 1 a for the RIL in selection set two, found that the high foliage symptom group (positive 47.9, negative 52.1) did not differ significantly (x^2^ = 6.411, df = 1, *p* = 0.423) from the low foliage symptom group (positive 41.8, negative 58.2).

**Figure 4:**
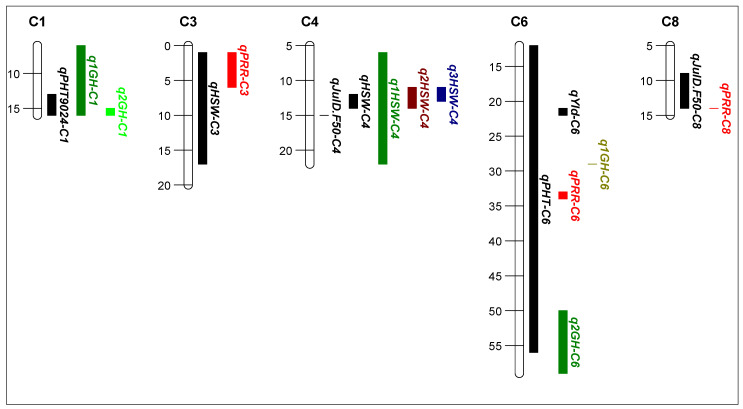
Position of quantitative trait loci (QTL) detected in the Yorker × *C. echinospermum* backcross recombinant inbred line (RIL) population (black bars) for the Tamworth 2017 non-diseased grain production experiment. The vertical QTL bars represent the physical position (two LOD drops from the QTL maximum likelihood value). The colored bars represent the QTL identified in previous studies. Growth habit *q1GH-C1* [20], *q1GH-C6* [26], *q2GH-C6* [20], phytophthora root rot resistance *qPRR-C3*, *qPRR-C6*, *qPRR-C8* [11], 100-seed weight *q1HSW-C4* [20], *q2HSW-C4* [25], and *q3HSW-C4* [24].

QTL analysis of the YB RIL population: from the whole population analysis, seven QTL were identified for traits that differed significantly (*p* < 0.05) among the 20 RIL high disease pressure phenotype groups (Table 1 b, Figure 2). The traits included two QTL each for 50% flowering, height, and 100-seed weight and one QTL for yield. The LOD scores ranged from 3.2 to 5.5, and the phenotypic variation explained by a single QTL ranged from 7.3% to 25.8%. Four of the seven QTL were associated with negative agronomic effects (one for later flowering, one for shorter plants, one for lower grain yield, and one for lighter seed) for alleles from the *C. echinospermum* backcross parent. The physical position of the QTL and the QTLs identified in previous studies in the same genomic regions are shown in Figure 4. A comparison of the frequency of the agronomically positive and negative QTL alleles as classified in Table 1 b for the RIL in selection set two found that the high foliage symptom group (positive 59.7, negative 40.3) differed significantly (x^2^ = 8.749, df = 1, *p* = 0.003) from the low foliage symptom group (positive 39.8, negative 60.2).

### 2.2. Phytophthora Inoculated and Natural Phytophthora Inoculum Experiments

This analysis tested if the foliage symptoms and yields of the low and high foliage symptom phenotypes in selection set one were related among experiments with different disease pressure under 2015 *P. medicaginis* inoculated and 2016 natural inoculum conditions. Across all the *P. medicaginis* inoculated field experiments, the presence of PRR symptoms was confirmed on susceptible genotypes through the expression of stem cankers and the isolation of *P. medicaginis* confirmed by morphology. The identity of the *P. medicaginis* cultures isolated from the natural inoculum experiment was also confirmed using a qPCR method [13].

For the RB RIL *P. medicaginis* inoculated experiments, there were significant (*p* < 0.05) differences among the two foliage symptom groups among all disease symptom (proportions of dead seedlings, dead podded plants, non-symptomatic podded plants, all podded plants, and AUDPS values) and yield (grain yield, seed number, 100-seed weight, and seed number/podded plant) parameters under both dryland and irrigated conditions, except for 100-seed weight under dryland conditions (Table 2 a). For the YB RIL, 100-seed weight and seed number per plant under dryland and the proportion of dead podded plants under irrigated were the only parameters to not differ significantly (*p* > 0.05) among the foliage symptom groups (Table 2 b).

The 2016 natural inoculum experiment occurred during an above average rainfall season and the results demonstrated the effects of high PRR disease pressure (Table 3). For example, for the very PRR susceptible var. Rupali, 100% died as seedlings. For the RB RIL, there were significant (*p* < 0.05) differences between the two foliage symptom groups for all four PRR symptom parameters (proportions of dead seedlings, dead podded plants, non-symptomatic podded plants, all podded plants) and grain yield. The parameters that differed significantly (*p* < 0.05) among the foliage symptom groups for the YB RIL were very similar to the other population, except that the proportion of podded plants (0.004–0.083) did not differ significantly (*p* > 0.05) among groups.

**Figure 5:**
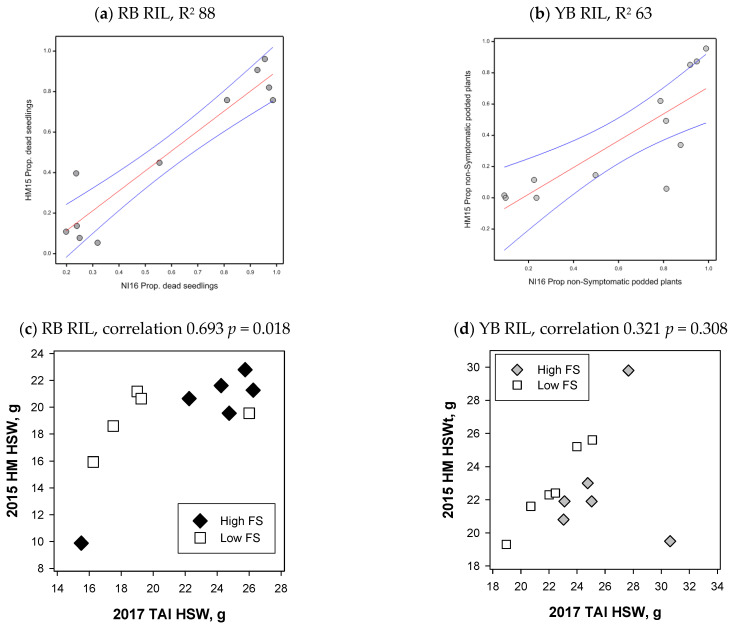
Foliage symptom (FS) and 100-seed weight comparisons. Symptom comparisons for selection group 1 (**a**) recombinant inbred line (RIL) from the cv. Rupali × *C. echinospermum* backcross (RB) for the proportion (Prop.) of dead seedlings, (**b**) Yorker × *C. echinospermum* backcross (YB) RIL comparing the proportion of non-symptomatic plants in the Tamworth 2016 natural inoculum experiment (NI16) and the Hermitage 2015 (HM15) *Phytophthora medicaginis* inoculated irrigated experiments, linear regression with red fitted regression lines with blue 95% confidence intervals. Hundred-seed weight comparisons of selection group 1 of the Tamworth 2017 non-diseased grain production experiment and the Hermitage 2015 *P. medicaginis* inoculated dryland experiments for the (**c**) RB RIL, correlation 0.693, *p* = 0.018 and the (**d**) YB RIL, correlation 0.321, *p* = 0.308, correlation with the high FS RIL C019 excluded 0.826, *p* = 0.0017.

Linear regression was used to compare the RB RIL PRR symptom and yield parameter values among the inoculated experiments and the natural inoculum experiment, the proportions of dead seedlings and podded plants both gave consistently high R^2^ values (>85) for the irrigated and dryland experiments (Table 4, Figure 5a). For the YB RIL, R^2^ values were lower than those of the other population, with the proportion of non-symptomatic plants with pods giving the highest value (R^2^ 63) across both the dryland and irrigated experiments (Table 4, Figure 5b). For that population, both the proportion of dead seedlings and the All Pod category gave the next highest set of R^2^ values (close to 50), which was consistent across both the irrigated and dryland experiments.

### 2.3. Foliage Symptom and Yield Parameters to Identify PRR High Resistance RIL with High Yield Potential

These analyses tested if the foliage symptom and yield parameters of RIL under *P. medicaginis* disease pressure could be used to identify high PRR resistance RIL with a high yield potential.

For selection group one RIL of both the RB and YB populations, the 100-seed weight did not differ between the low and high disease foliage symptoms groups in dryland *P. medicaginis* inoculated experiments, while the second yield component seed number did differ significantly between the foliage symptom groups (Table 2). For the RB RIL, the 100-seed weights of *P. medicaginis* inoculated RIL and RIL with no Phytophthora disease from the 2017 Tamworth experiment were positively and significantly correlated (0.693, *p* < 0.05) (Figure 5c). For the RB RIL, six RIL had 100-seed weights above 20 g in the dryland *P. medicaginis* inoculated experiment, five of these RIL had two beneficial QTL for 100-seed weight (Supplementary Material, Figure S2). For the 100-seed weight comparisons of all YB RIL, there was no significant relationship (0.321, *p* = 0.308), while the exclusion of a high foliage symptom RIL (C019), which had a 20g reduction in 100-seed weight under the PRR disease condition, provided a positive and significant correlation (0.826 *p* = 0.0017) (Figure 5d, Supplementary Material, Figure S3). For the YB RIL, it was notable that the two low foliage symptom RIL with the highest 100-seed weights in the dryland *P. medicaginis* inoculated experiment both had two beneficial QTL for 100-seed weight (Supplementary Material, Figure S3). Correlation of the 100-seed weights from the irrigated 2015 experiments against the 2017 experiment did not provide significant correlations (Supplementary Material Figures S2 and S3).

## 3. Discussion

We compared RIL from chickpea × *C. echinospermum* populations with contrasting partial resistance reactions to Phytophthora root rot (PRR). In the absence of Phytophthora root rot, for the most resistant RIL in the most resistant population, there was evidence of unintended trade-offs from *C. echinospermum*-based PRR resistance on agronomic traits for the low foliage symptom RIL in comparison to the high foliage symptom RIL. In addition, in the most PRR resistant population, the most PRR resistant RIL with low foliage symptoms had a higher frequency of alleles for the negative agronomic traits from the *C. echinospermum* backcross parent in comparison to the high foliage symptom RIL. Among the foliage symptom and yield parameter comparisons, survival to maturity was the most consistent parameter across all environments; in comparison, grain yield was more variable across environments, especially under irrigated conditions. In contrast to grain yield, the 100-seed weight of low and high foliage symptom RIL under moderate (dryland) disease pressure was significantly correlated with non-PRR diseased 100-seed weights. This yield component trait in conjunction with the 100-seed weight QTL information may be useful to identify RIL from PRR resistance phenotyping experiments for further seed number evaluations to identify high yield potential material.

It is important to evaluate if there are potential trade-offs in the material associated with disease resistance or tolerance [27]. We found evidence in a Phytophthora-free single-row RIL experiment for differences in phenology and yield-related traits for a group of 20 low foliage symptom RIL in comparison with 20 high foliage symptom RIL from the YB population. This differs for RB RIL, with lower levels of PRR resistance where only seed weights and plant heights differed. The other set of six RIL selected under low PRR disease pressure in 2014 did not provide consistent Phytophthora-free differences in grain production parameters, and phenotyping under high PRR pressure in 2015 showed that half of the low foliage symptom group had inconsistent resistance phenotype performance across environments. This finding confirms the importance of assessing quantitative resistance reactions across multiple environments [28,29]. Li et al. [16] identified an introgression segment on chromosome 3 from the *C. echinospermum* backcross, 04067-81-2-1-1-B, in the pedigree of the chickpea breeding lines that had lower grain yields than lines without the introgression segment when grown in Phytophthora-free conditions. Our findings related to 04067-81-2-1-1-B extend from the study of Li et al. [16] for the attribution of yield effects by providing evidence that: alleles for QTL affecting multiple agronomic traits on multiple chromosomes are provided from both parents and include both benefits (i.e., non-drag) and negative yield traits (i.e., drag) from 04067-81-2-1-1-B as a parent, which was consistent with the effects of transgressive segregation reported for crosses of *C. echinospermum* and chickpea [18,19,20]; QTL for the effects on phenology and yield were also co-located with PRR resistance QTL [11]; it is the RIL with high levels of PRR resistance that have significantly lower yields, rather than all material crossed with 04067-81-2-1-1-B; furthermore, linkage between PRR resistance and yield potential was demonstrated by low foliage symptom RIL from the population with the highest level of PRR resistance having a significantly higher frequency of alleles associated with agronomic drag effects from the *C. echinospermum* backcross parent than the high foliage symptom RIL from this population.

Partial resistance is based on a large number of QTL of small effect, which provides a continuous phenotype distribution [30,31]. We were able to demonstrate that traits such as 100-seed weight, plant height, and flowering traits on four separate and three separate chromosomes in two RIL populations, respectively, were co-located with PRR resistance QTL [11]. Further research is required to determine that the alleles from the 04067-81-2-1-1-B parent, negatively affecting the above traits, have come from the *C. echinospermum* parent (ILWC245) or from other genotypes in its pedigree. In addition, it will be important to determine if the mechanism can be attributed to linkage drag or pleiotropy, as selection strategies for breaking linkage drag effects, such as reducing the size of introgression segments, can be used to avoid upstream non-resistance region effects [32,33].

In addition to the negative effects on phenology and yield through introgression with *C. echinospermum*, there was also evidence of positive effects. Transgressive segregation describes the ability of loci controlling a trait to be affected both negatively and positively by alleles from each parent and has been widely reported to occur in crosses of chickpea with both *C. reticulatum* and *C. echinospermum* [17,18,19,20,34,35]. Wild relatives of pulse crops can provide novel sources of resistance that are not available in the target crop species gene pool, but wild relatives of both chickpea and lentil have also presented agronomic challenges due to later flowering, late maturity, and greater indeterminacy than the target crop [36,37,38]. In the case of chickpea PRR resistance breeding, the foliage symptom category group RIL interaction results for low foliage symptom YB RIL identified some individual high-resistance RIL with agronomically favorable traits, such as early flowering and podding. Here, transgressive segregation may provide opportunities for targeted selection of high PRR resistance RILs that also have agronomically favorable traits.

Understanding which parameters are the most consistent across a range of environments is important to inform selection strategies for both disease resistance and yield traits. A source of genotype by environment interactions in quantitative systems is differing infection intensities among genotypes [11,21,39]; thus, it was important to understand which parameters among phenological development, foliage symptoms, and the yields of selected chickpea × *C. echinospermum* RILs are the most consistently related across environments. This study identified complexities for PRR resistance assessments in chickpea × *C. echinospermum* derivatives in identifying parameters that accounted for the most variation across the environments. As for the RB population, the proportions of dead seedlings and podded plants were the best parameters, but for the YB population, the proportion of non-symptomatic RIL consistently accounted for the most variation. In addition, it was useful to identify that grain yield was a variable parameter across environments but in comparison a number of disease assessment parameters were less variable than grain yield.

Effective methods to identify material with both high partial resistance and high yield potentials are of importance to variety development breeding programs. The grain yields of PRR-infected grain legumes, such as chickpea and soybean, represent the ability of resistant material to survive and produce grain, as yield depends on a level of partial resistance [14,31,40]. Therefore, it may not be possible to separate the resistant material with the highest yield potential. To identify the parameters indictive of differential yield potential rather than disease resistance in the presence of disease, without the inclusion of disease-protected control treatments appears challenging. One route towards this goal may be through identifying traits that are the least affected by the disease relative to more disease sensitive traits. Seed weight in chickpea is a highly heritable trait [41,42,43,44]. In our dryland *P. medicaginis* inoculated experiments, 100-seed weight was evaluated as a candidate trait, as in contrast to another yield component seed number, 100-seed weight did not differ between the two foliage symptom groups, although significant reductions occurred under the higher disease pressure of irrigated conditions. From our relatively limited data set, the results indicated that under low PRR disease pressure, PRR-affected 100-seed weights were significantly related to non-PRR diseased 100-seed weights and that RIL with high levels of partial resistance and 100-seed weights could be identified. Further work is required to substantiate these initial findings, including evaluating genotype responses under multi-row rather than single-row plots and identifying lines with high seed numbers and favorable 100-seed weights for a high grain yield potential. Markers for 100-seed weight QTL may also assist with this process.

Linkages between the genetic basis of the important agronomic traits were identified to assist future breeding. A QTL analysis revealed a total of 26 QTL associated with key agronomic and yield traits in the RB population. Most of these QTL are in similar chromosomal regions as identified in previous studies. Hence, it is highly likely that these QTL are co-located with the QTL identified in previous studies based on their physical position and may contain candidate genes reported in earlier studies. However, further fine mapping is required to narrow down the QTL intervals and shortlist the potential candidate genes. Of the 26 QTL, 16 QTL are co-located with the PRR resistance QTL identified by Amalraj et al. [11] in the same mapping population. The Julian day 50% flowering time QTL on chromosome 3 (*qJulD.F50-C3*) is in the same region as a days to 50% flowering time QTL detected previously by Upadhyaya et al. [23] and reported to contain a *bZIP* (basic-leucine zipper) transcription factor, known for its role in controlling the flowering time [45,46,47]. The physical position of 100-seed weight QTL on chromosomes 3 and 7 is co-located with the 100-seed weight QTL detected by Barmukh et al. [24]. Likewise, the 100-seed weight QTL on chromosome 4 (*qHSW-C4*) has also been identified in the YB population and in multiple previous studies in the same genetic region [20,24,25]. The number of candidate genes with a role in controlling seed parameters, including a gene on chromosome 3 (pentatricopeptide repeat-containing protein) and five genes on chromosome 4 (tubby-like F-box protein 8, pentatricopeptide repeat-containing, pentatricopeptide repeat-containing protein, homoserine kinase, transcription factor bHLH118), have been reported in the *qHSW-C4* region [24]. The plant height QTL on chromosome 1 (*qPHT-C1*) is co-located with the growth habit (prostrate to erect) QTL identified by Lakmes et al. [20], whereas the *qPHT-C4* is co-located with the growth habit [26] and plant height QTL [24]. Numerous QTL clusters were also detected in the YB population. A region on chromosomes 3, 4, and 6 harbors multiple QTL for flowering and podding timing, indicating that the same genes could be responsible for controlling both traits. A recent study also found QTL clusters for these traits on chromosome 3 in interspecific populations but in different regions [35]. Also, the co-located plant height and 100-seed weight on chromosome 4 are in the same regions as identified by Barmukh et al. [24], indicating a genetic linkage between these two traits. In the YB population, three QTL on chromosomes 3 (*qHSW-C3*), 6 (*qPHT-C6*), and 8 (*qJulD.F50-C8*) are co-located with the PRR resistance QTL identified earlier in this mapping population [11]. The plant height QTL on chromosome 1 (*qPHT-C1*) is in the same genetic region as the growth habit QTL (prostrate to erect) identified in previous studies [20,48], and has been reported to contain two zinc finger genes as potential candidates in this region [48]. Also, a plant height QTL on chromosome 6 (*qPHT-C6*) previously detected in same region [20,26] contains a DNA ligase encoding gene, known to enhance plant growth and development [49]. This study has identified several genomic regions associated with important domestication and yield traits in two interspecific RIL mapping populations. The alleles of these traits can be introgressed into adapted varieties to improve their genetic gain for yield.

In summary, we found evidence that the yields of chickpea-*C. echinospermum* RIL in the absence of PRR disease differed among genotypes with low and high levels of PRR resistance, especially for the RIL population with the highest levels of PRR resistance, the effect was associated with later flowering and podding, lighter seed, and shorter plants in the low foliage symptom RILs. We identified the co-location of multiple PRR resistance and agronomic QTL and higher frequencies of alleles with negative effects for agronomic traits in the most PRR-resistant RIL population from the *C. echinospermum* backcross parent. Differences in PRR disease pressure affected foliage symptoms and yields among chickpea-*C. echinospermum* RIL across *P. medicaginis* in inoculated and naturally infected experiments. Despite differences in PRR disease pressure, a number of development and foliage symptom parameters were strongly correlated, these may be useful PRR resistance selection traits. Finally, in addition to identifying numerous useful QTL, we also identified that the 100-seed weight of material PRR phenotyped under moderate disease pressure may identify high yield component lines for later seed number evaluations.

## 4. Materials and Methods

The populations were phenotyped for grain production and yield-associated traits in experiments in the absence of PRR disease in 2017; in earlier experiments in 2015, the populations were phenotyped for PRR foliage symptom development in *P. medicaginis* inoculated experiments. The yields of the selected RIL were evaluated as part of the 2015 *P. medicaginis* inoculated experiments and a natural *P. medicaginis* inoculum field experiment in 2016. A summary of the number of RIL and key experimental factors for each of the field experiments is provided in the Supplementary Material Table S2.

### 4.1. RIL Development and Seed Sources

Two F6-derived RIL populations were developed by the National Chickpea Breeding Program. The *C. echinospermum* backcross line, D0467-1-81-2-1-1(B), with moderate PRR resistance, was crossed to the Australian chickpea cultivars ‘Rupali’ (very susceptible to PRR) and ‘Yorker’ (moderately susceptible to PRR) to form the RB and YB populations, respectively, as previously described by Amalraj et al. [11].

### 4.2. Non-Diseased Grain Production Experiments

To determine the RIL yields in the absence of PRR disease, two single-row yield assessment experiments were conducted at the Tamworth Agricultural Institute in 2017, with an experiment for each of the two RIL populations. This site was selected due to a history of the absence of PRR and during the experiment susceptible check entries and RIL parents were assessed for PRR symptoms. Each experiment consisted of four replicates in a randomized complete block design, each plot was a 1.2 m single row, with 179 RIL entries for the RB RIL population and 180 RIL entries for the YB RIL population. The plots were sown on May 12 and 13 using the methods described by Amalraj et al. [11], but without the application of the *P. medicaginis* inoculum.

All the plants in each plot were included in the phenology assessments, the 50% flower and 50% podding dates were recorded when half of the plants in a plot fulfilled these criteria. The plots were assessed over a 60-day period from mid-August to mid-October two to three times per week depending on the extent of the development observed each week. The flowering and pod development times were recorded in Julian days. At plant senescence on November 20, the height of the tallest plant in each plot was measured in mm with a ruler from the ground surface to the growing tip of the tallest growth point. In December, the plants in each plot were hand harvested, the plants were threshed, and the grain yields and 100-seed weights were determined.

The 2017 winter–spring growing season in Tamworth had four consecutive months following planting of below average rainfall for June (49 mm), July (20 mm), August (21 mm), and September (10 mm), followed by above average rainfall in October (90 mm) (Supplementary Material Table S3). The mean minimum temperatures during the August (1.2 °C) to September (4.5 °C) chickpea flowering period were below average in both August (2.8 °C) and September (5.8 °C) 2017.

### 4.3. Quantitative Trait Locus (QTL) Analysis

A QTL analysis was carried out on the phenotypic data from the 2017 Tamworth experiments and the genetic linkage maps developed by Amalraj et al. [11]. Composite interval mapping (CIM) was performed using WinQTLCart-Version 2.5 (Model 6 standard analysis with five control markers and a window size of 10 cm) [50]. The log of odds (LOD) value thresholds were determined with 1000 times permutations [51] at a 1-cM walk speed (*p* = 0.05). To find the physical position of the QTL identified in this study and compare it with the previously reported QTL, all the marker sequences within the region of a two LOD drop from the maximum likelihood value were searched using BLASTn against the CDC Frontier genome assembly v1 [52]. The map graphics and QTL positions were drawn using MapChart 2.1 [53]. A Chi-squared test was performed to compare the frequencies of the alleles with a positive and negative between RIL in the high and low foliage symptom groups in selection set two.

### 4.4. Phytophthora Inoculated and Natural Phytophthora Inoculum Experiments

#### Hermitage 2014 and 2015 RIL *P. medicaginis* Inoculated Experiments

The methods used for the 2014 and 2015 RIL *P. medicaginis* inoculated field experiments at the Hermitage Research Facility site (−28.204908 S, 152.102689 E) are described by Amalraj et al. [11].

The experimental plots were sown with a four-row seeder with separate in-furrow delivery of the in-solution *Mesorhizobium ciceri* rhizobia inoculant and the 10 isolate *P. medicaginis* mixture at sowing. Each plot had 20 seeds per single 1.2 m row plot. Each experiment contained four replicates in a randomized block design, check varieties (n = 6) covering a PRR resistance spectrum were included. The site had a deep, self-mulching, grey vertisol [54]. In 2014, there was no in-crop irrigation, while in 2015, supplementary irrigation was applied to two of the four experiments with dripper tape (T-tape, Rivulas Irrigation, Israel), with applications delivered for 7 to 8 h/day over a 2–3-day period, depending on the volume of the application (Supplementary Material Table S3). The other two 2015 experiments were managed as dryland.

Rainfall and air temperature records were accessed from the Bureau of Meteorology (BOM) (station number 055325), located at the Hermitage Research Facility less than 1 km from the experimental sites. For the 2014 season, 97 mm of the in-crop rainfall was received during the field experiments. For the 2015 season, the monthly rainfall was below the long-term average for July, September, and October, which triggered the application of supplementary irrigation during September and October (Supplementary Material Table S3). Above average rainfall in November 2015 led to a total of 160 mm of in-crop rainfall. The mean minimum temperatures during August and September (the chickpea flowering period) were above average in August 2014 and close to average in September 2014, but for 2015, the mean minimum temperatures were below average for August and September.

For each experiment in each plot, following the completion of emergence, the number of seedlings was counted. A minimum of three disease assessments were then made; firstly, when the early foliage symptoms were evident in susceptible check varieties; the second and final assessments were made at mid-season and late season. At each assessment, separate counts of the number of chlorotic, dead, and total number of plants were made. The late-season assessments were conducted before plant senescence, and the counts of plants with premature senescence were also made at the final assessment. At the final assessment, the phenological development of the dead plants was categorized as: produced no pods (died as seedlings prior to flowering) or as podded, and the counts of each category were made. At this assessment, counts were also made of the number of chlorotic, senescent, and healthy non-senesced plants.

### 4.5. Selection of RIL Phenotype Groups from Hermitage 2014 and 2015 RIL Experiment Results

The selection of two sets of RIL foliage symptom groups to account for the environmental effects on resistance phenotype expression was made for comparing the yield and agronomic traits of the RIL phenotype groups in later experiments.

Selection set one, the low disease pressure RIL foliage symptom groups: from the 2014 Hermitage experiments, the GLMM output for the backtransformed proportion of plants that had died at the end of the season assessment was used as the criteria to select the groups, each containing six high and six low foliage symptom RIL, as described in Bithell et al. [14] in more detail. Briefly, in each population from the group of foliage symptom that had nil plant death (RB RIL, n = 42; YB RIL, n = 84), six lines were randomly selected as low foliage symptom lines using a random number function in excel (Microsoft Office Standard 2016). From the group of lines that had more than 30% of dead plants for the RB RIL (n = 25) and 10% for the YB RIL (n = 15) populations, six lines were randomly selected from each as high disease lines using the same method. These 12 RIL in each population and the RIL parents in each of their respective dryland and irrigated Warwick 2015 experiments had all plants in each plot harvested by hand after physiological maturity. The yields were determined as described earlier.

Selection set two, the high disease pressure RIL foliage symptom groups: the 2015 irrigated Hermitage experiments had a higher PRR disease pressure than the 2014 and 2015 dryland experiments [11]. The 2015 irrigated results were used for a second selection set for RIL with differing foliage symptom phenotypes. As with selection set one, the GLMM output for the backtransformed proportion of plants that had died at the final end of the season assessment in each population in the 2015 irrigated experiments was used as the criteria. This final proportional death criteria was then used to rank all RIL, and the 20 RIL with the lowestt plant death and the 20 RIL with the highest plant death were selected as the respective low and high foliage symptoms from each of the RB and YB RIL populations.

### 4.6. Tamworth 2016 Natural Inoculum Experiment

To assess the foliage symptoms and yield traits under natural inoculum conditions, an experiment was sown into an area of naturally occurring *P. medicaginis* inoculum in 2016, where large losses of Kabuli chickpeas to PRR occurred in the 2015 season at the Tamworth Agricultural Institute (−31.147395 S, 150.983151 E). The site had a vertisol soil type with a medium clay content (54%) [55]. The area was not large enough for complete RIL population screening, the 24 RIL in selection set one (2014 season selection) from the two populations (RB and YB RIL) and their parents, 10 RIL low foliage symptom group RIL from selection set two (4 RB RIL and 6 YB RIL) and check varieties (n = 7) were planted on May 24, 2016 in a randomized complete block design with 6 replicates. The experiment was sown with a four-row seeder with separate in-furrow delivery of in-solution *Mesorhizobium ciceri* rhizobia. Each plot consisted of 33 seeds in a 1.2 m single row. Using counts of all plants in each plot at each assessment plant establishment was recorded after emergence and disease assessments were made by counting chlorotic and dead plants, including those with and without pods. On December 8, each plot was hand harvested for grain, and yields determined as described earlier.

Rainfall and air temperature records were accessed from the Bureau of Meteorology (BOM) (station number 055325), located at Tamworth Airport, which was 12 km from the experiment. The 2016 winter–spring growing season in Tamworth had high rainfall in June (169 mm), August (83 mm), and September (125 mm) (Supplementary Material Table S3). For the 6 months from June to November, there was a total of 494 mm of rainfall, which was 229 mm higher than the long-term (1993 to 2021) median rainfall for this period. The mean minimum temperatures during the August to September chickpea flowering period were above average in both August and September.

### 4.7. Design and Analyses

All the experiments were designed using DiGGer ver. 1.0.2 [56]. The experiments that included check varieties were supra-replicated on a block and sub-block basis. The residuals were examined, and if necessary, data were appropriately transformed to meet the requirements for the residuals to be normally distributed. All the statistical analyses of the plot data were carried out using GenStat 19th edition [57].

Non-diseased grain production experiments, Tamworth 2017: For the analysis of the timing of the RIL flower and pod development and the grain parameters in both interspecific populations, a GLMM with a Poisson log distribution link and a Wald test were used. The differences in the development and grain parameters between the high and low RIL foliage symptom groups in selection set one and two were evaluated using an ANOVA for RIL nested within the foliage symptom group.

Hermitage 2015 RIL *P. medicaginis* inoculated experiments: To provide a multi-assessment-based disease parameter, the area under the disease progress stairs (AUDPS) [58] was calculated as separate parameters for the proportion of dead plants per plot and symptomatic (chlorotic plus dead) plants per plot for the disease assessments for each experiment. Using three hourly temperature records from a soil depth of 100 mm recorded 750 m from the field experiment (BOM Station # 41525), the thermal time accumulation (^°^Cd) was calculated using a T_b_ of 5 °C and T_max_ of 35 °C. The daily accumulation was calculated for the daily maximum and minimum temperature values between 5 °C and 35 °C. To test for differences in the disease symptoms and grain parameters between the high and low RIL in the foliage symptom groups in selection set one, a GLMM of RIL nested within the foliage symptom group was performed, a binominal distribution logit link was used for the proportional data and a Poisson log distribution link used for the non-proportional data.

Tamworth 2016 natural inoculum experiment: Similar to the above analyses of disease symptoms and grain parameters between the high and low RIL in the foliage symptom groups in selection set one, a GLMM of RIL nested within the foliage symptom group was used.

Hermitage 2015 and Tamworth 2016 experiment comparisons: To compare the RIL performance in the four Hermitage 2015 experiments to the same RIL in the Tamworth natural inoculum experiment, a linear regression of the backtransformed logit values was used with the natural inoculum data as the explanatory variable and the Hermitage data as the response variable.

Evaluation of disease and yield parameters to identify RIL with high PRR resistance and yield potential: To identify which development, disease, or yield parameter in the PRR resistance phenotyping experiments may be used to identify high PRR resistance RIL with a high yield potential, correlations of the results from the four Hermitage 2015 *P. medicaginis* inoculated experiments to yield results for these RIL in the Tamworth 2017 non-diseased yield experiments were conducted.

## Figures and Tables

**Table 1:** Quantitative trait locus (QTL) for the Tamworth 2017 non-diseased grain production experiments with significant differences between foliage symptom groups for phenology (flowering and podding timing) and yield (grain weight and 100-seed weight) related trait results for the (a) Rupali × *Cicer echinospermum* backcross (RB) and (b) Yorker × *C. echinospermum* backcross (YB) recombinant inbred line (RIL) populations.

**(a) RB RIL**								
**Trait**	**QTL**	**Chr**	**Position**	**Genetic Map Distance**	**Physical Position Mbp**	**LOD**	**Additive Effect**	***R*** **^2^**
Plant Height (mm)	*qPHT-C1*	C1	74.8	72.6–77.0	40.49–48.30	3.5	20.63	5.6
Plant Height (mm)	*qPHT-C3*	C3	5.5	0.0–9.6	0.17–6.42	5.4	26.79	9.0
Plant Height (mm)	*qPHT-C5*	C5	90.9	83.0–99.9	33.27–47.31	6.4	−36.35 *	15.8
Plant Height (mm)	*qPHT-C4*	C4	68.3	53.8–74.0	9.10–12.66	7.1	28.96	10.9
100-seed weight (HSW)	*qHSW-C3*	C3	22.9	0.02–42.9	0.17–29.59	4.6	−1.25 *	9.7
100-seed weight (HSW)	*qHSW-C7*	C7	33.6	24.2–45.8	3.64–8.77	7.3	−1.15 *	8.1
100-seed weight (HSW)	*qHSW-C4*	C4	72.8	68.2–74.0	11.86–12.66	33.2	2.94	52.8
**(b) YB RIL**								
**Trait**	**QTL**	**Chr**	**Position**	**Genetic map distance**	**Physical position Mbp**	**LOD**	**Additive effect**	***R*** **^2^**
Julian Day 50% flowering	*qJulD.F50-C4*	C4	94.4	92.4–99.0	14.7–15.45	5.5	−3.06	12.1
Julian Day 50% flowering	*qJulD.F50-C8*	C8	109.2	97.2–123.8	8.76–14.25	5.0	1.69 *	13.8
Plant Height (mm)	*qPHT-C1*	C1	148.3	133.0–180.0	12.80–16.48	4.7	−31.25 *	25.8
Plant Height (mm)	*qPHT-C6*	C6	122	111.6–128.1	12.52–56.17	5.5	21.36	12.0
Yield (g)	*qYld-C6*	C6	101.2	96.4–105.7	20.56–22.18	3.6	11.52	8.8
100-seed weight (HSW)	*qHSW-C3*	C3	12.7	0.0–59.1	1.00–16.65	3.4	−0.79 *	7.3
100-seed weight (HSW)	*qHSW-C4*	C4	88.2	82.1–90.8	12.32–14.45	3.2	0.83	7.5

Trait name, QTL name, chromosome number (Chr), QTL peak position (cM), genetic map distance (cM), physical position (Mbp) based on CDC Frontier genome assembly v1, log of odds (LOD), additive effect, and phenotypic variation (*R*^2^) explained by the QTL (% variation) are shown. Positive (Rupali or Yorker) and negative (04067-81-2-1-1) values indicate the parent responsible for increasing the relative value; traits where the beneficial allele (i.e., taller plant or earlier flowering) comes from the introgression line are indicated by an *.

**Table 2:** Hermitage 2015 *Phytophthora medicaginis* inoculated experiments, with two recombinant line (RIL) populations, (a) Rupali × *Cicer echinospermum* backcross (RB) and (b) Yorker × *C. echinospermum* backcross (YB) in four experiments managed as dryland or irrigated, with values for RIL parents and the sets of low foliage (LFS) and high foliage symptom (HFS) RIL for the parameters: proportion dead seedlings (Dead Se.), proportion dead plants with pods (Dead Pod), proportion non-symptomatic plants with pods (Non Sym.Pod), proportion of all dead or live plants with pods (All Pod), area under the disease progress stairs (AUDPS), grain yield (g/m^2^), 100-seed weight (100 SW, g), total seed number (Seed no., no./m^2^) and seed number/all podded plants (Seed/PodP, no./m^2^), the number of plots with nil seed included as a superscript ^0^ in the yield column. The first superscript value (^1^ = probability < 0.001, ^2^ = probability < 0.01, ^ns^ = probability > 0.05) is the significance of the Wald statistic of the main effects between the LFS and HFS groups, a second superscript is the significance of the symptom group*RIL interactions for non-proportional results only.

**(a) RB RIL**									
**Dryland**	**Dead Se.**	**Dead Pod**	**No-Sym.Pod**	**All Pod**	**AUDPS**	**Yield**	**100SW**	**Seed No.**	**Seed/PodP**
Rupali	0.725	0.150	0.105	0.275	0.490	54.4 ^01^	20.2	248	61.9
04067-81-2-1-1(B)	0	0.016	0.254	1.0	0.004	403.9	21.6	1871	116.6
LFS group	0.099	0.027	0.407	0.901	0.066	277.4	19.2	1466	99.1
HFS group	0.693	0.138	0.151	0.307	0.405	49.5 ^02^	19.3	217	40.1
Wald statistic	40.8 ^1^	12.0 ^1^	6.64 ^2^	40.8 ^1^	68.3 ^1,2^	87.6 ^1,2^	16.6 ^ns,ns^	112.5 ^1,2^	24.8 ^1,ns^
**Irrigated**	**Dead Se.**	**Dead Pod**	**No-Sym.Pod**	**All Pod**	**AUDPS**	**Yield**	**100SW**	**Seed No.**	**Seed No./** **PodP**
Rupali	0.849	0.151	0	0.151	0.577	5.1	8.5	35	18.5
04067-81-2-1-1(B)	0	0.149	0.601	1.0	0	400.1	19.2	2058	137.4
LFS group	0.154	0.536	0.085	0.846	0.305	170.1	15.3	1096	71.5
HFS group	0.777	0.220	0.002	0.223	0.552	11.2 ^03^	12.0	73	14.1
Wald statistic	66.5 ^1^	17.7 ^1^	8.65 ^2^	66.5 ^1^	152.8 ^1,1^	109.2 ^1,1^	4.3 ^2,1^	151.0 ^1,1^	78.5 ^1,ns^
**(b) YB RIL**									
**Dryland**	**Dead Se.**	**Dead Pod**	**No-Sym.Pod**	**All Pod**	**AUDPS**	**Yield**	**100SW**	**Seed No.**	**Seed No./** **PodP**
Yorker	0.328	0.246	0.213	0.639	0.370	160.9	21.7	735	77.6
04067-81-2-1-1(B)	0	0	1.0	1.0	0	273.7	22.1	1240	76.9
LFS group	0.028	0.015	0.897	0.973	0.033	330.4	22.7	1451	91
HFS group	0.439	0.166	0.259	0.563	0.340	162.1 ^01^	22.8	693	68
Wald statistic	29.5 ^1^	16.3 ^1^	30.6 ^1^	29.1 ^1^	22.1 ^1,ns^	30.1 ^1,1^	0.25 ^ns,2^	32.3 ^1,2^	2.57 ^ns,2^
**Irrigated**	**Dead Se.**	**Dead Pod**	**No-Sym.Pod**	**All Pod**	**AUDPS**	**Yield**	**100SW**	**Seed No.**	**Seed No./** **PodP**
Yorker	0.448	0.463	0.090	0.552	0.533	35.4	15.7	225	31.0
04067-81-2-1-1(B)	0.015	0	0.985	0.985	0.008	376.5	19.6	1904	121.9
LFS group	0.113	0.198	0.682	0.880	0.157	233.9	17.8	1261.4	80.8
HFS group	0.749	0.191	0.055	0.246	0.535	19.5 ^05^	14.0	115.0	27.8
Wald statistic	77.6 ^1^	0.01 ^ns^	41.2 ^1^	79.6 ^1^	43.5 ^1,2^	54.8 ^1,1^	6.11 ^2,2^	68.6 ^1,2^	7.56 ^2,ns^

**Table 3:** Tamworth 2016 natural inoculum experiment, with selected low foliage (LFS) and high foliage symptom (HFS) lines of two recombinant inbred line (RIL) populations, Rupali × *C. echinospermum* backcross (RB) and Yorker × *C. echinospermum* backcross (RB) RIL, for the parameters of final proportions of dead seedlings (Dead Se.), dead plants with pods (Dead Pod), non-symptomatic plants with pods (Non Sym.Pod), dead and alive plants with pods (All Pod), and grain yield (g/m^2^), the number of plots with nil seed produced is included as a superscript ^0^ in the yield column for (a) the three parents of the two RIL populations and (b) the average parameter values for each foliage symptom group in each population. Wald statistic for differences among RIL, ^1^ = probability < 0.001, ^ns^ = probability > 0.05.

**(a) parents**					
	**Dead Se.**	**Dead Pod**	**No-Sym.Pod**	**All Pod**	**Yield**
Rupali (A)	1.0	0	0	0	0 ^06^
Yorker (A)	0.366	0.063	0.553	0.620	254.1
04067-81-2-1-1(B)	0.053	0	0.948	0.947	469.7
**(b) RIL groups**					
**RB RIL**	**Dead Se.**	**Dead Pod**	**No-Sym.Pod**	**All Pod**	**Yield**
LFS	0.249	0.149	0.580	0.732	200.3
HFS	0.868	0.031	0.091	0.121	56.6 ^016^
Wald statistic	176.4 ^1^	64.5 ^1^	101.0 ^1^	184.8 ^1^	86.7 ^1^
**YB RIL**	**Dead Se.**	**Dead Pod**	**No-Sym.Pod**	**All Pod**	**Yield**
LFS	0.089	0.004	0.888	0.892	432.5
HFS	0.574	0.083	0.327	0.404	163.9 ^05^
Wald statistic	128.0 ^1^	16.4 ^ns^	133.2 ^1^	122.1 ^1^	94.9 ^1^

**Table 4:** Hermitage 2015 *Phytophthora medicaginis* inoculated experiments versus the Tamworth 2016 natural inoculum single-row experiment results. The percentage variance (R^2^ values) of linear regression of backtransformed values for the 2015 dryland or irrigated experiments with all low and high disease lines (df = 11) in each of two recombinant line (RIL) populations, Rupali × *C. echinospermum* backcross (RB) and Yorker × *C. echinospermum* backcross (YB), against the same entries in the 2016 natural inoculum experiment. The parameters were the final proportions of dead seedlings (Dead Se.), dead plants with pods (Dead Pod), non-symptomatic plants with pods (Non Sym.Pod), dead and alive plants with pods (All Pod), and grain yield (g/m^2^). nf = no fit.

Populations	Dead Se.	Dead Pod	No-Sym.Pod	All Pod	Yield
RB RIL					
Dryland	88.4	5.5	43.3	86.6	55.3
Irrigated	88.1	nf	53.4	85.1	24.5
YB RIL					
Dryland	46.4	64.1	63.0	48.4	48.3
Irrigated	52.5	nf	63.0	52.6	34.3

## Data Availability

Data is available by request following permission from co-owners from the corresponding author.

## Supplementary Materials

The following supporting information can be downloaded at: https://www.mdpi.com/article/10.3390/plants13111432/s1, Figure S1: Tamworth 2017 non-diseased grain production experiment, trait correlations for all recombinant inbred lines in the (a) Rupali × *C. echinospermum* backcross and (b) Yorker × *C. echinospermum* backcross populations for the trats 50% flowering (F50), 50% podding (P50), Grain yield, Height and 100 seed weight; Supplementary information S1—Table S1: Quantitative trait loci (QTL) for the Tamworth 2017 non-diseased grain production experiment for the Rupali × *C. echinospermum* backcross recombinant inbred line (RIL) population for traits that did not differ significantly (*p* > 0.05) among the foliage symptoms groups. Table S2: Summary of field experiments with site, year, population indicated as cv. Rupali × *C. echinospermum* backcross (R-Ce) and cv. Yorker × *C. echinospermum* backcross population (Y-Ce), the number of recombinant inbred lines (RIL) in each experiment, inoculation of *Phytophthora medicaginis* is indicated where * indicates that the site had natural *P. medicaginis* inoculum, experiments where irrigation was applied in-crop are labelled as dryland and irrigated if irrigation was applied in-crop, and the four experiments where data was used to make RIL selection sets are indicated; Table S3: Monthly values for mean minimum daily air temperature (°C), monthly rainfall total (mm), including long term average (LTA) and in-crop total rainfall (sowing to harvest dates) for field experiments at the Hermitage Research Facility (HRF) from 2014–2015 and Tamworth Agricultural Institute (TAI) in 2016–2017. Sources, TAI Bureau of Meteorology station 55325, HRF Bureau of Meteorology station 41525. For planting and harvest months, complete months rainfall (x) are presented followed by rainfall total after sowing or before harvest dated (y), presented as x/y. Superscript numbers provide supplementary irrigation amounts and timing in days after sowing (DAS); Figure S2: Selection set one RIL from the Rupali × *C. echinospermum* backcross populations for high and foliage symptom comparisons of the Tamworth 2017 non-diseased grain production experiment (2017 TAI) and Hermitage 2015 *Phytophthora medicaginis* inoculated (2015 HM) experiment results, (a) irrigated experiment grain yields, (b) irrigated experiment 100 seed weight (HSW) values and (c) dryland experiment 100 seed weight (correlation 0.693 P = 0.018) including the RIL code names and the number of agronomically beneficial 100 seed weight QTL indicated as -0 (no QTL), -1 (one QTL), -2 (2 QTL) identified for each RIL, ni = no information for QTL mapping; Figure S3: Selection set one RIL from the Yorker × *C. echinospermum* backcross populations for high and foliage symptom comparisons of the Tamworth 2017 non-diseased grain production experiment (2017 TAI) and Hermitage 2015 *Phytophthora medicaginis* inoculated (2015 HM) experiment results, (a) irrigated experiment grain yields, (b) irrigated experiment 100 seed weight (HSW) values and (c) dryland experiment 100 seed weight (correlation 0.321 *p* = 0.308, correlation with the high foliage RIL C019 excluded 0.826 P 0.0017) including the RIL code names (blue text for high foliage symptom RIL, black text for low foliage symptoms RIL) and the number of agronomically beneficial 100 seed weight QTL indicated as -0 (no QTL), -1 (one QTL), -2 (2 QTL) identified for each RIL, ni = no information for QTL mapping.

## References

[1] Savary S., Ficke A., Aubertot J.-N., Hollier C. (2012). Crop losses due to diseases and their implications for global food production losses and food security. Food Secur..

[2] Vock N.T., Langdon P.W., Pegg K.G. (1980). Root Rot of Chickpea Caused by Phytophthora Megasperma Var. Sojae in Queensland. Australas. Plant Pathol..

[3] Knights E.J., Açıkgöz N., Warkentin T., Bejiga G., Yadav S.S., Sandhu J.S., Yadav S.S., Redden R.J., Chen W., Sharma B. (2007). Area, production and distribution. Chickpea Breeding & Management.

[4] Salam M.U., Davidson J.A., Thomas G.J., Ford R., Jones R.A.C., Lindbeck K.D., MacLeod W.J., Kimber R.B.E., Galloway J., Mantri N. (2011). Advances in winter pulse pathology research in Australia. Australas. Plant Pathol..

[5] Singh K.B., Malhotra R.S., Halila M.H., Knights E.J., Verma M.M. (1993). Current status and future strategy in breeding chickpea for resistance to biotic and abiotic stresses. Euphytica.

[6] Dorrance A.E., McClure S.A. (2001). Beneficial Effects of Fungicide Seed Treatments for Soybean Cultivars with Partial Resistance to *Phytophthora sojae*. Plant Dis..

[7] Knights E.J., Southwell R.J., Schwinghamer M.W., Harden S. (2008). Resistance to Phytophthora medicaginis Hansen and Maxwell in wild Cicer species and its use in breeding root rot resistant chickpea (*Cicer arietinum* L.). Aust. J. Agric. Res..

[8] Schwinghamer M.W., Southwell R.J., Moore K.J., Knights E.J., Chen W., Sharma H.C., Muehlbauer F.J. (2011). Phytophthora Root Rot of Chickpea. Compendium of Chickpea and Lentil Diseases and Pests.

[9] Brinsmead R.B. (1985). Resistance in Chickpea to *Phytophthora megasperma* f. sp. medicaginis. Plant Dis..

[10] Dale M., Irwin J. (1991). Glasshouse and field screening of chickpea cultivars for resistance to Phytophthora megasperma f.sp. medicaginis. Aust. J. Exp. Agric..

[11] Amalraj A., Taylor J., Bithell S., Li Y., Moore K., Hobson K., Sutton T. (2018). Mapping resistance to Phytophthora root rot identifies independent loci from cultivated (*Cicer arietinum* L.) and wild (*Cicer echinospermum* P.H. Davis) chickpea. Theor. Appl. Genet..

[12] Knights T., Moore K., Cummings G. Yorker Desi Chickpea. Pulse Variety Management Package. https://www.pulseaus.com.au/storage/app/media/crops/chickpea/2009_VMP-Dchickpea-Yorker.pdf.

[13] Bithell S., Moore K., Herdina, McKay A., Harden S., Simpfendorfer S. (2020). Phytophthora root rot of chickpea: Inoculum concentration and seasonally dependent success for qPCR based predictions of disease and yield loss. Australas. Plant Pathol..

[14] Bithell S.L., Drenth A., Backhouse D., Harden S., Hobson K. (2023). Inoculum production of Phytophthora medicaginis can be used to screen for partial resistance in chickpea genotypes. Front. Plant Sci..

[15] Li H., Rodda M., Gnanasambandam A., Aftab M., Redden R., Hobson K., Rosewarne G., Materne M., Kaur S., Slater A.T. (2015). Breeding for biotic stress resistance in chickpea: Progress and prospects. Euphytica.

[16] Li Y., Ruperao P., Batley J., Edwards D., Martin W., Hobson K., Sutton T. (2022). Genomic prediction of preliminary yield trials in chickpea: Effect of functional annotation of SNPs and environment. Plant Genome.

[17] Singh M., Rani S., Malhotra N., Katna G., Sarker A. (2018). Transgressive segregations for agronomic improvement using interspecific crosses between *C. arietinum* L. × *C. reticulatum* Ladiz. and *C. arietinum* L. × *C. echinospermum* Davis species. PLoS ONE.

[18] Singh M., Kumar T., Sood S., Malhotra N., Rani U., Singh S., Singh I., Bindra S., Kumar S., Kumar S. (2022). Identification of promising chickpea interspecific derivatives for agro-morphological and major biotic traits. Front. Plant Sci..

[19] Sari D., Sari H., Eker T., Ikten C., Uzun B., Toker C. (2022). Intraspecific versus interspecific crosses for superior progeny in *Cicer* species. Crop. Sci..

[20] Lakmes A., Jhar A., Penmetsa R.V., Wei W., Brennan A.C., Kahriman A. (2022). Inheritance of seed weight and growth habit in 10 intercross chickpea (*Cicer arietinum*) nested association mapping populations. Plant Breed..

[21] Price J.S., Bever J.D., Clay K. (2004). Genotype, environment, and genotype by environment interactions determine quantitative resistance to leaf rust (*Coleosporium asterum*) in *Euthamia graminifolia* (Asteraceae). New Phytol..

[22] Upadhyaya H.D., Dwivedi S.L., Sharma S., Varshney R.K., Thudi M., Muehlbauer F.J. (2017). Managing and Discovering Agronomically Beneficial Traits in Chickpea Germplasm Collections. Chickpea Genome.

[23] Upadhyaya H.D., Bajaj D., Das S., Saxena M.S., Badoni S., Kumar V., Tripathi S., Gowda C.L.L., Sharma S., Tyagi A.K. (2015). A genome-scale integrated approach aids in genetic dissection of complex flowering time trait in chickpea. Plant Mol. Biol..

[24] Barmukh R., Soren K.R., Madugula P., Gangwar P., Shanmugavadivel P.S., Bharadwaj C., Konda A.K., Chaturvedi S.K., Bhandari A., Rajain K. (2021). Construction of a high-density genetic map and QTL analysis for yield, yield components and agronomic traits in chickpea (*Cicer arietinum* L.). PLoS ONE.

[25] Singh V.K., Khan A.W., Jaganathan D., Thudi M., Roorkiwal M., Takagi H., Garg V., Kumar V., Chitikineni A., Gaur P.M. (2016). QTL-seq for rapid identification of candidate genes for 100-seed weight and root/total plant dry weight ratio under rainfed conditions in chickpea. Plant Biotechnol. J..

[26] Upadhyaya H.D., Bajaj D., Srivastava R., Daware A., Basu U., Tripathi S., Bharadwaj C., Tyagi A.K., Parida S.K. (2017). Genetic dissection of plant growth habit in chickpea. Funct. Integr. Genom..

[27] Dwivedi S.L., Reynolds M.P., Ortiz R. (2021). Mitigating tradeoffs in plant breeding. iScience.

[28] Kulkarni R.N. (1982). Environment as the Cause of Differential Interaction between Host Cultivars and Pathogenic Races. Phytopathology.

[29] Pariaud B., Ravigné V., Halkett F., Goyeau H., Carlier J., Lannou C. (2009). Aggressiveness and its role in the adaptation of plant pathogens. Plant Pathol..

[30] St. Clair D.A. (2010). Quantitative Disease Resistance and Quantitative Resistance Loci in Breeding. Annu. Rev. Phytopathol..

[31] Wang H., Martin S.K.S., Dorrance A.E. (2012). Comparison of Phenotypic Methods and Yield Contributions of Quantitative Trait Loci for Partial Resistance to *Phytophthora sojae* in Soybean. Crop. Sci..

[32] Lewis R.S., Linger L.R., Wolff M.F., Wernsman E.A. (2007). The negative influence of N-mediated TMV resistance on yield in tobacco: Linkage drag versus pleiotropy. Theor. Appl. Genet..

[33] Li J., Chitwood J., Menda N., Mueller L., Hutton S.F. (2017). Linkage between the I-3 gene for resistance to Fusarium wilt race 3 and increased sensitivity to bacterial spot in tomato. Theor. Appl. Genet..

[34] Nagpal S., Sirari A., Sharma P., Singh S., Mandahal K.S., Singh H., Singh S. (2023). Marker trait association for biological nitrogen fixation traits in an interspecific cross of chickpea (*Cicer arietinum* × *Cicer reticulatum*). Physiol. Mol. Biol. Plants.

[35] Newman T.E., Jacques S., Grime C., Mobegi F.M., Kamphuis F.L., Khentry Y., Lee R., Kamphuis L.G. (2023). Genetic dissection of domestication traits in interspecific chickpea populations. Plant Genome.

[36] Gorim L.Y., Vandenberg A. (2017). Evaluation of Wild Lentil Species as Genetic Resources to Improve Drought Tolerance in Cultivated Lentil. Front. Plant Sci..

[37] Robertson L., Ocampo B., Singh K. (1997). Morphological variation in wild annual Cicer species in comparison to the cultigen. Euphytica.

[38] Croser J.S., Ahmad F., Clarke H.J., Siddique K.H.M. (2003). Utilisation of wild Cicer in chickpea improvement—Progress, constraints, and prospects. Aust. J. Agric. Res..

[39] Dorrance A.E., McClure S.A., Martin S.K.S. (2003). Effect of Partial Resistance on Phytophthora Stem Rot Incidence and Yield of Soybean in Ohio. Plant Dis..

[40] Tooley P.W. (1984). The Relationship between Rate-Reducing Resistance to Phytophthora megasperma f. sp. glycinea and Yield of Soybean. Phytopathology.

[41] Hossain S., Ford R., McNeil D., Pittock C., Panozzo J.F. (2010). Inheritance of seed size in chickpea (*Cicer arietinum* L.) and identification of QTL based on 100-seed weight and seed size index. Aust. J. Crop Sci..

[42] Ton A., Anlarsal A.E. (2017). Estimation of genetic variability for seed yield and its components in chickpea (*Cicer arientinum* L.) genotypes. Legum. Res. Int. J..

[43] Ali M.A., Nawab N.N., Abbas A., Zulkiffal M., Sajjad M. (2009). Evaluation of selection criteria in *Cicer arietinum* L. using correlation coefficients and path analysis. Aust. J. Crop Sci..

[44] Wang R., Gangola M.P., Jaiswal S., Gaur P.M., Båga M., Chibbar R.N. (2017). Genotype, environment and their interaction influence seed quality traits in chickpea (*Cicer arietinum* L.). J. Food Compos. Anal..

[45] Abe M., Kobayashi Y., Yamamoto S., Daimon Y., Yamaguchi A., Ikeda Y., Ichinoki H., Notaguchi M., Goto K., Araki T. (2005). FD, a bZIP Protein Mediating Signals from the Floral Pathway Integrator FT at the Shoot Apex. Science.

[46] Kawamoto N., Sasabe M., Endo M., Machida Y., Araki T. (2015). Calcium-dependent protein kinases responsible for the phosphorylation of a bZIP transcription factor FD crucial for the florigen complex formation. Sci. Rep..

[47] Nan H., Cao D., Zhang D., Li Y., Lu S., Tang L., Yuan X., Liu B., Kong F. (2014). GmFT2a and GmFT5a Redundantly and Differentially Regulate Flowering through Interaction with and Upregulation of the bZIP Transcription Factor GmFDL19 in Soybean. PLOS ONE.

[48] Ali L., Azam S., Rubio J., Kudapa H., Madrid E., Varshney R.K., Castro P., Chen W., Gil J., Millan T. (2015). Detection of a new QTL/gene for growth habit in chickpea CaLG1 using wide and narrow crosses. Euphytica.

[49] Waterworth W.M., Masnavi G., Bhardwaj R.M., Jiang Q., Bray C.M., West C.E. (2010). A plant DNA ligase is an important determinant of seed longevity. Plant J..

[50] Wang S., Basten C., Zeng Z. (2012). Windows QTL Cartographer 2.5.

[51] Churchill G.A., Doerge R.W. (1994). Empirical threshold values for quantitative trait mapping. Genetics.

[52] Varshney R.K., Song C., Saxena R.K., Azam S., Yu S., Sharpe A.G., Cannon S., Baek J., Rosen B.D., Tar’An B. (2013). Draft genome sequence of chickpea (*Cicer arietinum*) provides a resource for trait improvement. Nat. Biotechnol..

[53] Voorrips R.E. (2002). MapChart: Software for the graphical presentation of linkage maps and QTLs. J. Hered..

[54] Thomson S.J., Cameron J.A.L., Dalal R.C., Hoult E. (2007). Alternate wet-dry regime during fallow failed to improve nitrogen release from added legume residues in legume-wheat rotations on a Vertisol. Aust. J. Exp. Agric..

[55] Schwenke G.D., Haigh B.M. (2019). Can split or delayed application of N fertiliser to grain sorghum reduce soil N2O emissions from sub-tropical Vertosols and maintain grain yields?. Soil Res..

[56] Coombes N. DiGGer: DiGGer Design Generator under Correlation and Blocking. R Package Version 1.0.0, 201. http://nswdpibiom.org/austatgen/software/.

[57] Anon GenStat Committee (2018). The Guide to GenStat Release 19.1.

[58] Simko I., Piepho H.-P. (2012). The Area under the Disease Progress Stairs: Calculation, Advantage, and Application. Phytopathology.

