# Supplementary material for: Selection for Phytophthora Root Rot Resistance in Chickpea Crosses Affects Yield Potential of Chickpea × Cicer echinospermum Backcross Derivatives
Source: Plants (Basel). 2024 May 22;13(11):1432. doi: 10.3390/plants13111432 (PMC11174912; doi:10.3390/plants13111432)
Supplement: Supplementary file 1 [file plants-13-01432-s001.zip › plants-2970130-supplementary.pdf]

Supplementary material

a) Rupali × *C. ech.* backcross b) Yorker × *C. ech.* backcross RIL

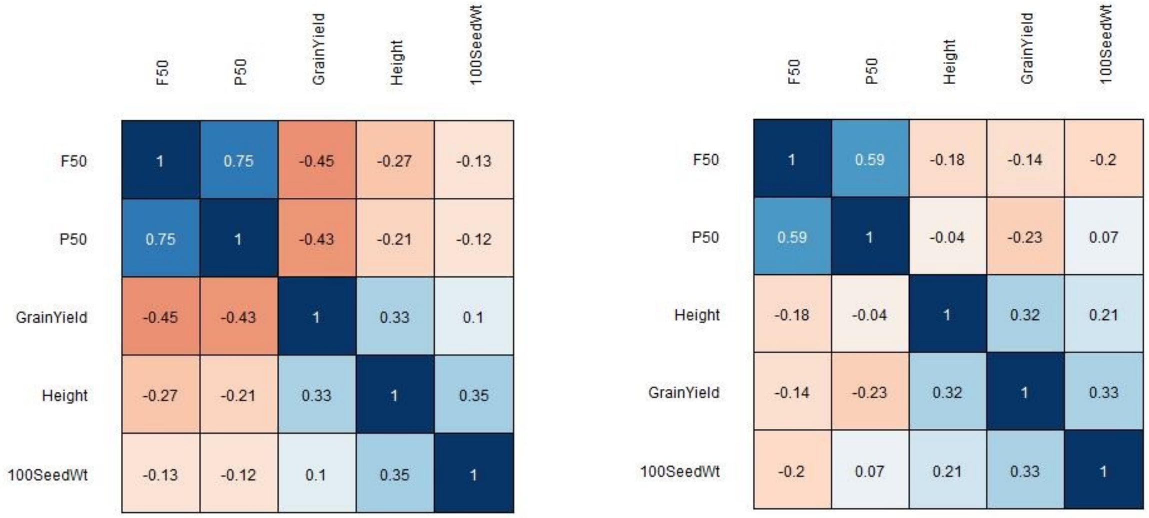

Figure S1. Tamworth 2017 non-diseased grain production experiment, trait correlations for all recombinant inbred lines in the a) Rupali × *C. echinospermum* backcross and b) Yorker × *C. echinospermum* backcross populations for the trats 50% flowering (F50), 50% podding (P50), Grain yield, Height and 100 seed weight.

Supplementary information, S1

RB RIL Yield Traits between High and Low Foliage Symptom Phenotypes

Selection set one, 6 RILs low disease pressure phenotype groups: There was no significant difference in the grain yields (P = 0.225) among the 6 RIL high foliage and the 6 RIL low foliage symptom groups, although there was a significant (P < 0.001, LSD 47.20) symptom group by RIL interaction. However, 100 seed weights differed significantly (P < 0.001, LDS 0.623) with the high foliage (19.19 g) symptom group having a lower seed weight than the low foliage (23.02 g) symptom groups, there was also a significant (P < 0.001, LSD 1.526) symptom group by RIL interaction. For this selection set there was also significantly (P< 0.05) earlier 50% flowering and 50% podding for the high foliage symptom (50% flowering 260.4 Julian day, 50% podding 275.0 Julian day) than the low foliage symptom group (50% flowering 262.5 Julian day LSD 1.747, 50% podding 276.8 Julian day LSD 1.375), both of these parameters also provided significant (P < 0.01) symptom group by RIL interactions. Plant height significantly (P < 0.001, LSD 35.88) differed between groups, with the low foliage symptom (570 mm) group having shorter plants than the high foliage symptom (666 mm) group.

YB RIL Yield Traits between High and Low Foliage Symptom Phenotypes

Selection set one, 6 RIL low disease pressure phenotype groups: The 6 RIL high foliage symptom group (237.6 g) had significantly (P < 0.001, LSD 22.09) higher yields than the 6 RIL low foliage symptom group (184.0 g) and there was a significant (P = 0.001, LSD 54.11) symptom group by RIL interaction. In addition, the 100 seed weight values for the 6 RIL high foliage symptom group was significantly (P < 0.001, LSD 0.725) higher (25.7 g) than the low foliage symptom group (22.2 g) and there was a significant (P < 0.001, LSD 1.776) symptom group by RIL interaction. However, between the 6 RIL groups there was no significant difference in 50% flowering dates (P = 0.205). For the 50% podding dates the high foliage symptom groups (Julian day 303.2) had significantly (P < 0.001, LSD 1.047) later 50% podding than the low foliage symptom group (Julian day 300.9). Plant height also differed significantly (P = 0.002, LSD 32.24) between groups, with the low foliage symptom (569 mm) group having shorter plants than the high foliage symptom (621 mm) group included a significant (P < 0.001, LSD 76.51) foliage symptom group by RIL interaction.

Table S1. Quantitative trait loci (QTL) for the Tamworth 2017 non-diseased grain production experiment for the Rupali × *C. echinospermum* backcross recombinant inbred line (RIL) population for traits that did not differ significantly (P > 0.05) among the foliage symptoms groups.

**Trait**	**QTL**	**Chr**	**Position**	**Genetic map distance**	**Physical position**	**LOD**	**Additive effect**	R^2
Julian Day first flower	*qJulD.FF-C3*	C3	90.7	81.7-110.6	34,626,842-39,954,271	3.3	0.84	9.1
Julian Day first flower	*qJulD.FF-C6*	C6	117	112.0-122.5	26,349,074-54,278,408	2.8	-0.66	5.8
Julian Day first flower	*qJulD.FF-C4*	C4	15.4	5.1-16.6	1,301,528-3,230,430	5.3	-1.35	13.3
Julian Day 50% flowering	*qJulD.F50-C3*	C3	64.7	55.7-75.4	29,640,141-34,468,002	4.3	0.92	8.1
Julian Day 50% flowering	*qJulD.F50-C8*	C8	62	49.9-75.0	14,248,306-15,860,322	3.2	0.75	5.8
Julian Day 50% flowering	*qJulD.F50-C6.1*	C6	87.5	86-97.2	30,945,455-56,116,038	3.3	0.88	6.0
Julian Day 50% flowering	*qJulD.F50-C6.2*	C6	119.3	113.3-122.5	26,349,074-54,278,408	5.3	-1.12	9.8
Days to 50% flower from first flower	*qDF50FFF-C3*	C3	5.5	0.4-23.9	167,197-6,416,914	3.4	-0.65	6.8
Days to 50% flower from first flower	*qDF50FFF-C5*	C5	66.4	58.1-73.9	41,313,593-46,294,343	4.9	0.82	10.2
Julian Day first pod	*qJulD.FP-C1*	C1	64.6	63.4-66.4	23,044,539-46,635,567	2.9	0.64	5.5
Julian Day first pod	*qJulD.FP-C3*	C3	66.9	65.1-94.7	30,838,580-39,136,825	4.2	0.78	8.0
Julian Day first pod	*qJulD.FP-C8*	C8	30.4	29.3-45.9	5,630,020-8,756,483	5.1	-0.85	9.1
Julian Day first pod	*qJulD.FP-C4*	C4	6	0.02-18.8	218,547-3,997,490	3.6	-0.72	7.0
Julian Day 50% pod	*qJulD.P50-C3*	C3	66.9	55.7-75.4	29,640,141-34,468,002	7.5	1.11	13.9
Julian Day 50% pod	*qJulD.P50-C8*	C8	41	30.6-48.9	5,783,554-8,756,483	4.0	-0.77	7.0
Julian Day 50% pod	*qJulD.P50-C6.1*	C6	87.5	86.0-97.2	30,945,455-56,116,038	2.9	0.64	5.0
Julian Day 50% pod	*qJulD.P50-C6.2*	C6	119.3	112.0-122.5	26,349,074-54,278,408	3.7	-0.82	5.9
Julian Day 50% pod	*qJulD.P50-C4*	C4	14.4	6.4-20.7	1,469,997-4,206,856	3.7	-0.80	7.7
Yield	*qYld-C5*	C5	93.9	85.6-105.9	33,270,980-47,311,525	5.4	-17.76	18.6

Trait name, QTL name, chromosome number (Chr), QTL peak position (cM), genetic map distance (cM), physical position (bp) based on CDC Frontier genome assembly v1, log of odds (LOD), additive effect and phenotypic variation (R^2) explained by the QTL (% variation) are shown. Positive (Yorker) and negative (04067-81-2-1-1) values indicate the parent responsible for increasing the phenotypic value.

Table S2. Summary of field experiments with site, year, population indicated as cv. Rupali × *C. echinospermum* backcross (R-Ce) and cv. Yorker × *C. echinospermum* backcross population (Y-Ce), the number of recombinant inbred lines (RIL) in each experiment, inoculation of *Phytophthora medicaginis* is indicated where * indicates that the site had natural *P. medicaginis* inoculum, experiments where irrigation was applied in-crop are labelled as dryland and irrigated if irrigation was applied in-crop, and the four experiments where data was used to make RIL selection sets are indicated.

Site	Year	Population	No. RIL	Inoculum	Dryland/Irrigated	note
Hermitage	2014	R-Ce	181	Y	dryland	Results used to make selection set 1
Hermitage	2014	Y-Ce	165	Y	dryland	Results used to make selection set 1
Hermitage	2015	R-Ce	192	Y	dryland	
Hermitage	2015	R-Ce	193	Y	irrigated	Results used to make selection set 2
Hermitage	2015	Y-Ce	175	Y	dryland	
Hermitage	2015	Y-Ce	176	Y	irrigated	Results used to make selection set 2
Tamworth	2016	R-Ce/Y-Ce	16/18	Y*	dryland	
Tamworth	2017	R-Ce	179	N	dryland	
Tamworth	2017	Y-Ce	180	N	dryland	

Table S3. Monthly values for mean minimum daily air temperature (C°), monthly rainfall total (mm), including long term average (LTA) and in-crop total rainfall (sowing to harvest dates) for field experiments at the Hermitage Research Facility (HRF) from 2014-2015 and Tamworth Agricultural Institute (TAI) in 2016-2017. Sources, TAI Bureau of Meteorology station 55325, HRF Bureau of Meteorology station 41525. For planting and harvest months, complete months rainfall (x) are presented followed by rainfall total after sowing or before harvest dated (y), presented as x/y. Superscript numbers provide supplementary irrigation amounts and timing in days after sowing (DAS).

**HRF**	**Year**	**Jan**	**Feb**	**Mar**	**Apr**	**May**	**Jun**	**Jul**	**Aug**	**Sep**	**Oct**	**Nov**	**Dec**	**In-crop**
Mean minT	2014	16.8	16.7	15.6	11.8	7.9	5.1	0.4	6.0	6.6	10.9	16.2	16.8	
Mean minT	2015	17.6	16.4	16.4	10.6	6.8	5.4	3.0	3.1	5.3	10.3	15.2	15.3	
	LTA	17.1	17.1	15.4	11.5	7.1	4.9	3.1	3.3	7.1	10.7	13.7	15.9	
Rainfall mm	2014	31.0	8.8	170.2	8.0	22.0	13.0	6.8	46.3	23.2	14.4	33.0	214.2	97
Rainfall mm	2015	101	56.8	52	48.9	74	19.4/18.2	21.8	24.8	10.8 [1]	20.2 [2]	125.8/64.8	93.6	160.6
	LTA	83.7	64.3	62.5	31.2	41.2	34.9	26	24.3	34.2	68.8	88.9	105.9	
**TAI**	**Year**	**Jan**	**Feb**	**Mar**	**Apr**	**May**	**Jun**	**Jul**	**Aug**	**Sep**	**Oct**	**Nov**	**Dec**	**In-crop**
Mean minT	2016	17.0	16.1	15.7	12.2	6.6	6.1	3.7	3.2	7.2	7.1	10.1	16.9	
Mean minT	2017	19.6	18.6	15.1	9.0	6.3	3.5	-0.1	1.2	4.5	11.4	11.9	16.5	
	LTA	17.6	16.9	14.5	10.0	6.0	3.6	2.3	2.8	5.8	9.7	13.2	15.6	
Rainfall mm	2016	99.6	0.8	22.0	4.8	60.8	169.0	28.6	83.2	132.6	76.0	11.6	96.6	494
Rainfall mm	2017	125.4	18.6	124.2	13.8	60.8	48.6	19.8	20.6	10.0	89.8	64.4	39.4	332
	LTA	60.2	68.4	49.9	24.3	29.5	52.7	40.3	38.1	43.7	54.6	81.2	75.4	

[1]65 mm 92-93 DAS, [2]105 mm 128-129 DAS of supplementary irrigation applied over a 48 h period. HM irrigation only applied to irrigated not dryland experiments.

a) Rupali × *C. ech.* backcross, irrigated

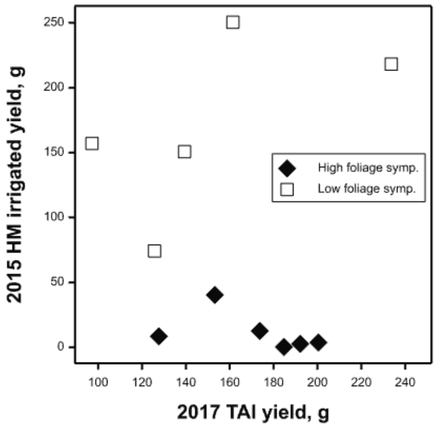

b) Rupali × *C. ech.* backcross RIL, irrigated

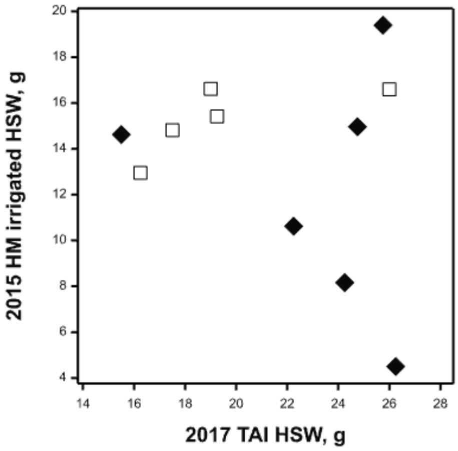

c) Rupali × *C. ech.* backcross, dryland

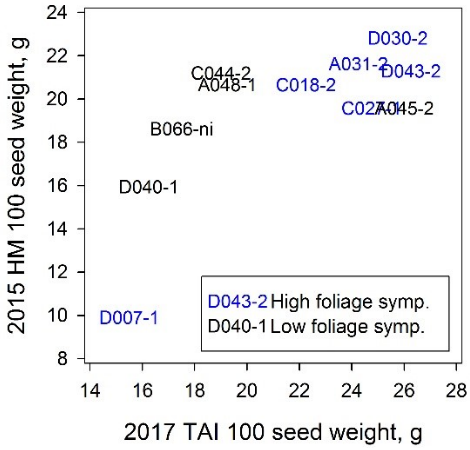

Figure S2. Selection set one RIL from the Rupali × *C. echinospermum* backcross populations for high and foliage symptom comparisons of the Tamworth 2017 non-diseased grain production experiment (2017 TAI) and Hermitage 2015 *Phytophthora medicaginis* inoculated (2015 HM) experiment results, a) irrigated experiment grain yields, b) irrigated experiment 100 seed weight (HSW) values and c) dryland experiment 100 seed weight (correlation 0.693 P = 0.018) including the RIL code names and the number of agronomically beneficial 100 seed weight QTL indicated as -0 (no QTL), -1 (one QTL), -2 (2 QTL) identified for each RIL, ni = no information for QTL mapping.

a) Yorker × *C. ech.* backcross, irrigated

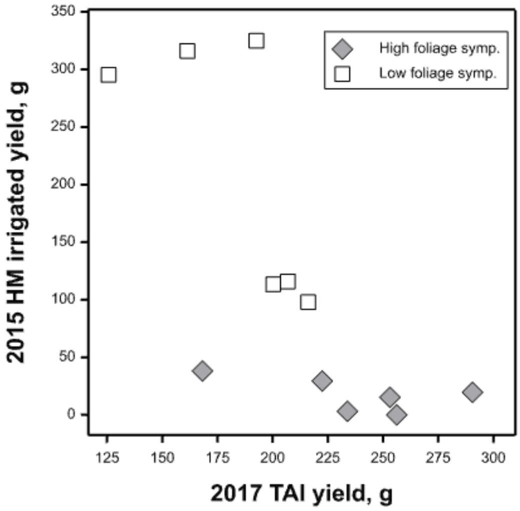

b) Yorker × *C. ech.* backcross RIL, irrigated

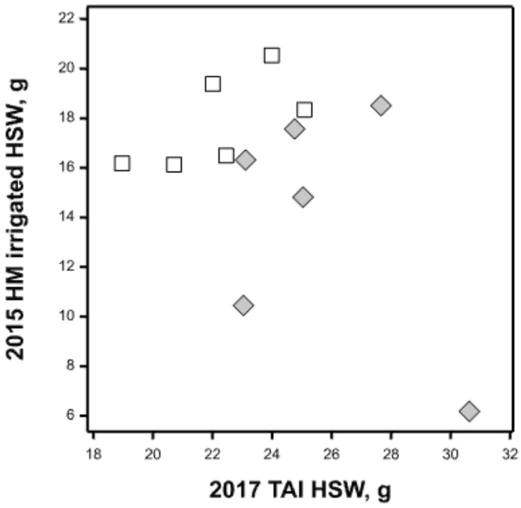

c) Yorker × *C. ech.* backcross, dryland

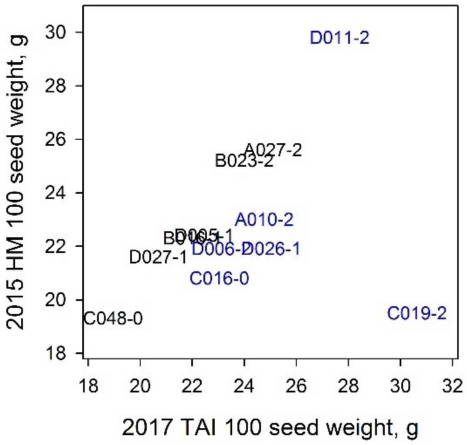

Figure S3. Selection set one RIL from the Yorker × *C. echinospermum* backcross populations for high and foliage symptom comparisons of the Tamworth 2017 non-diseased grain production experiment (2017 TAI) and Hermitage 2015 *Phytophthora medicaginis* inoculated (2015 HM) experiment results, a) irrigated experiment grain yields, b) irrigated experiment 100 seed weight (HSW) values and c) dryland experiment 100 seed weight (correlation 0.321 P = 0.308, correlation with the high foliage RIL C019 excluded 0.826 P 0.0017) including the RIL code names (blue text for high foliage symptom RIL, black text for low foliage symptoms RIL) and the number of agronomically beneficial 100 seed weight QTL indicated as -0 (no QTL), -1 (one QTL), -2 (2 QTL) identified for each RIL, ni = no information for QTL mapping.
